# Seroepidemiology of Herpes Simplex Viruses Type 1 and 2 in Pregnant Women in Croatia

**DOI:** 10.3390/medicina60020284

**Published:** 2024-02-07

**Authors:** Tatjana Vilibic-Cavlek, Marko Belamaric, Thomas Ferenc, Dan Navolan, Branko Kolaric, Ljiljana Milasincic, Ljiljana Antolasic, Mateja Vujica Ferenc, Maja Vilibic, Adriana Lukunic, Maja Bogdanic

**Affiliations:** 1 Department of Virology, Croatian Institute of Public Health, 10000 Zagreb, Croatia; ljiljana.milasincic@hzjz.hr (L.M.); ljiljana.antolasic@hzjz.hr (L.A.); 2 School of Medicine, University of Zagreb, 10000 Zagreb, Croatia; 3 Teaching Institute for Emergency Medicine, 10000 Zagreb, Croatia; mbelamaric123@gmail.com; 4 Department of Diagnostic and Interventional Radiology, University Hospital Merkur, 10000 Zagreb, Croatia; thomas.ferenc95@gmail.com; 5 Department of Obstetrics and Gynecology, ‘Victor Babes’ University of Medicine and Pharmacy, 300041 Timisoara, Romania; navolan@umft.ro; 6 Andrija Stampar Teaching Institute of Public Health, 10000 Zagreb, Croatia; branko.kolaric@stampar.hr; 7 Department of Social Medicine and Epidemiology, Medical Faculty, University of Rijeka, 51000 Rijeka, Croatia; 8 Department of Obstetrics and Gynecology, University Hospital Centre Zagreb, 10000 Zagreb, Croatia; matejavujica1@gmail.com; 9 Department of Psychiatry, Sestre Milosrdnice University Hospital Center, 10000 Zagreb, Croatia; maja.vilibic@gmail.com; 10 School of Medicine, Catholic University of Croatia, 10000 Zagreb, Croatia; 11 Department of Microbiology, University of Applied Health Sciences, 10000 Zagreb, Croatia; adriana.lukunic5@gmail.com

**Keywords:** herpes simplex virus, pregnant women, seroprevalence, epidemiology, Croatia

## Abstract

*Background and Objectives*: Herpes simplex viruses (HSV-1 and HSV-2) are one of the most widespread causes of human viral infections. In Croatia, only two published studies have analyzed the seroprevalence of HSV infections in childbearing-aged and pregnant women (2005–2010), while more recent data are lacking. This study aimed to analyze the prevalence and risk factors for HSV-1 and HSV-2 infections among pregnant women in Croatia in the period from 2011 to 2021. *Materials and Methods*: This study included 667 pregnant women aged 16–45 years submitted for HSV-1 and HSV-2 serology testing. Serum samples were initially screened for HSV-1 and HSV-2 IgM and IgG antibodies using a commercial ELISA test with a confirmation of HSV-2-positive samples using an immunoblot assay. *Results*: The overall IgG seroprevalence rates were 69.9% for HSV-1 and 3.8% for HSV-2. A significant gradual increase in the HSV-2 seroprevalence with age was observed from 0.5% in participants under 30 years to 8.3% in participants above 40 years. The HSV-1 seroprevalence was stable up to 40 years (70.0 and 68.3%, respectively), with an increase to 86.1%, but this difference did not reach statistical significance. Area of residence (urban or suburban/rural), geographic region (continental or coastal), and obstetric history (normal pregnancy or unfavorable obstetric history) were not associated with HSV-1 and HSV-2 seroprevalence. Older age was found to be a significant risk factor for HSV-2 seropositivity in both univariate and multivariate risk analysis. *Conclusions*: HSV-1 infection is widely prevalent among pregnant women with a stable trend over time. However, a declining trend in the HSV-2 seroprevalence was observed compared to 2005–2010. Serological screening in pregnant women is important in identifying seronegative women who are susceptible to HSV infection as well as seropositive women who are at risk for genital herpes recurrence during delivery.

## 1. Introduction

Herpes simplex viruses type 1 (HSV-1) and 2 (HSV-2), are double-stranded, enveloped DNA viruses that are members of the *Orthoherpesviridae* family, genus *Simplexvirus* [1]. HSV-1 and HSV-2 share 40–50% sequence homology of their genome structure [2]. The virion comprises a core, icosahedral capsid, tegument, and envelope, which contains viral glycoproteins that have essential roles in attachment to cellular receptors and virus entry into the host cells [1]. Among 13 glycoproteins, only the G glycoproteins of HSV-1 (gG-1) and HSV-2 (gG-2) induce type-specific B-cell responses [3].

Herpesviruses are one of the most common and widespread causative agents of human viral infections [4]. HSV-1 infections usually occur during childhood and adolescence and are transmitted via direct contact with infected oral secretions. Less commonly, HSV-1 can infect the genital area through oral–genital contact and cause genital herpes. HSV-2 infections are almost always transmitted through sexual contact [5]. After infecting the host, herpesviruses cause latent and persistent infection in the neuronal ganglia, where they can periodically reactivate. Symptomatic and asymptomatic viral shedding is common for both HSV-1 and HSV-2 [6].

Clinical presentation of HSV-1 infections is usually mild and, in most cases, consists only of ulcerations or blisters at the site of the infection, but can be severe with more frequent recurrences in immunocompromised patients. In rare cases, encephalitis and keratitis may occur [5]. However, HSV-2 infections pose a serious medical concern among women of reproductive age since the virus can be transmitted to fetuses and neonates. Primary HSV infection can be more severe in pregnant women than in non-pregnant women [7]. Maternal infection with HSV-1 or HSV-2 can occur at any stage of pregnancy. For HSV infections that occur in the last trimester of pregnancy, the risk of neonatal infection ranges from 30% to 50%, while it is only 1% for infections that occur in early pregnancy. Approximately 85% of perinatal infections occur during delivery, while transplacental transmission of HSV from mother to fetus is less common [2]. Both primary and recurrent maternal infections can cause congenital disease, but the risk after recurrent infection is low. Neonatal herpes is more common (50%) in infants born to mothers with a primary HSV infection than in infants born to mothers with recurrent HSV (<3%) [7].

Genital HSV infections during pregnancy are associated with spontaneous abortion, intrauterine growth retardation, preterm birth, and congenital and neonatal herpes. In infants infected intrapartum or postnatally, herpesvirus infections may present as disease localized to the skin, eye, and/or mouth, HSV encephalitis, or disseminated HSV infection that manifests as severe multiorgan dysfunction with a mortality of more than 80% in untreated patients [8]. Of the affected neonates, 50% have localized infections, 33% have central nervous system (CNS) involvement, and 17% have disseminated infections [7].

It has been estimated that half a billion of the world’s population have genital infection with HSV-2 or HSV-1, and several billion have oral HSV-1 infection [4]. Epidemiological studies conducted among childbearing-aged and pregnant women conducted in the 1990s and 2000s showed that the seroprevalence of HSV in Europe varies markedly between countries. In some countries, especially in multiethnic ones, regional differences within the same country were also observed [9,10,11]. Recent data on the HSV epidemiology in these population groups are missing.

Diagnostic methods for detecting HSV infections include direct (virus isolation, molecular diagnostics) and indirect methods (serology). The virus can be cultivated from swabs or needle aspirates in different types of cell cultures causing a cytopathic effect. This method requires high-quality sample collection, with proper handling and transportation of the sample. Molecular methods include polymerase chain reaction (PCR) and loop-mediated isothermal amplification (LAMP), which are very sensitive and specific in detecting the virus. Serologic methods include enzyme-linked immunoassays (ELISA), chemiluminescence assays (CLIA), and indirect immunofluorescence assays (IFA). Due to possible serological cross-reactivity, the Western blot (WB) is still considered the “gold standard” serological method for the detection of type-specific HSV antibodies and the differentiation between HSV-1 and HSV-2 [12].

There is no cure for HSV infection, except for some antiviral drugs that can reduce the symptom severity. Since neonatal herpes is one of the most severe complications of genital herpes, timely diagnosis and appropriate treatment of acute and recurrent episodes of HSV reduce the risk of vertical transmission and, in turn, the consequences for the neonate [2]. Despite the availability of antiviral drugs, the outcome of neonatal HSV infections remains poor, particularly for babies with disseminated multi-organ infections or CNS involvement [7].

In Croatia, there are few published studies on HSV prevalence in women of reproductive age. Between 2005 and 2009, a study on the seroprevalence of TORCH pathogens was conducted in 502 childbearing-aged women, showing a seroprevalence rate for HSV-1 and HSV-2 of 78.7% and 6.8%, respectively. IgM prevalence was 1.2% for both HSV-1 and HSV-2 [13]. From 2008 to 2010, a seroepidemiological study on herpesviruses was conducted in the Croatian general population. In a group of 530 pregnant women, 70.6% were seropositive to HSV-1 and 8.5% to HSV-2 [14]. The HSV seroprevalence was not studied thereafter.

This study aimed to analyze the prevalence and possible risk factors for HSV-1 and HSV-2 infections among pregnant women in Croatia. Since pregnant women represent a high-risk population for HSV infections and more recent data on herpesviruses’ epidemiology are lacking, the results of this study will contribute to a better understanding of the HSV epidemiology in this population group. In the context of possible severe HSV-2 disease sequelae, these results emphasize the importance of developing an HSV-2 vaccine as a key strategy to combat this infection [15].

## 2. Materials and Methods

### 2.1. Study Participants

**Figure 1:**
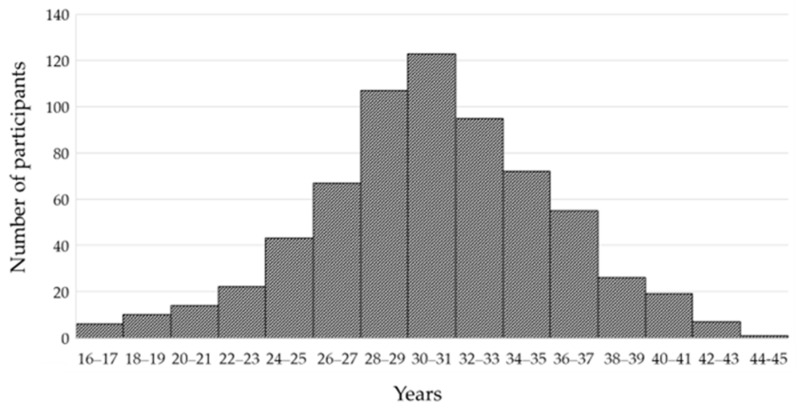
Distribution of study participants according to age.

The study included 667 pregnant women tested in the period from 2011 to 2021 (Figure 1). All samples from pregnant women submitted for HSV-1 and HSV-2 serology testing at the Croatian Institute of Public Health, the largest public health institute in the country, were included in the study. Study participants were from 19/21 Croatian counties. The distribution of participants by age is presented in Figure 1.

According to age, three groups were defined to best match the reported data: <30 years (*n* = 214; 32.1%), 30–39 years (*n* = 417; 62.5%), and ≥40 years (*n* = 36; 5.4%). The area of residence was urban in 614 (92.1%) participants and suburban/rural in 53 (7.9%) participants. Regarding the geographic region, 483 (72.4%) participants were from continental and 184 (27.6%) from coastal regions. Analyzing the obstetric history, 453 (67.9%) of participants had a normal pregnancy and 214 (32.1%) had an unfavorable obstetric history (habitual abortions, intrauterine growth retardation, and stillbirths).

### 2.2. Methods

Initial serological screening for HSV-1 and HSV-2 IgM and IgG antibodies was performed using a commercial enzyme immunoassay (ELISA; Virotech Diagnostics, Dietzenbach, Germany). The test uses the type-specific recombinant glycoproteins G, gG-1, and gG-2, which allows the differentiation between HSV-1 and HSV-2. In the ELISA test, the antibodies present in the serum form immune complexes with the antigen bound on the microtiter plate. Unbound immunoglobulins are removed by a washing process. The enzyme conjugate attaches to this complex. Unbound conjugates are removed by the next washing process. After adding the tetramethylbenzidine substrate solution, the bound horseradish peroxidase enzyme produces a blue dye. The color changes to yellow after adding the citrate-stopping solution. The optical density was read using a spectrophotometer (BioSan Microplate photometer, Riga, Latvia) at wavelength 450 nm and 620 nm reference filters within 10 min after stopping the reaction. The results were expressed in Virotech units (VE) and interpreted as follows: <9 negative; 9–11 borderline; >11 positive. The manufacturer states a diagnostic sensitivity of 98% for HSV-1 and 100% for HSV-2 and a specificity of 100% for HSV-1 and 95% for HSV-2.

Due to a possible cross-reactivity, HSV-1 IgM-positive and HSV-2 IgM- and/or IgG-positive samples were confirmed using a commercial WB (Euroline-WB, Euroimmun, Lübeck, Germany). The immunoblot assay detects type-specific HSV-1 and HSV-2 antibodies against gG-2. The test contains strips with electrophoretically separated antigens of HSV-1 (glycoproteins gC-1 and gG-1) and purified HSV-2 gG-2. The first step involves incubating the blot strips with patient serum samples. Specific antibodies in the serum bind to the antigens. To detect bound antibodies, alkaline phosphatase-labeled anti-human antibodies catalyze a color reaction after adding a nitroblue tetrazolium chloride/5-bromo-4-chloro-3 indolylphosphate substrate solution. After stopping the reaction using distilled water and drying, the strips were scanned and evaluated with EUROLineScan (Euroimmun, Lübeck, Germany). The test shows a sensitivity and specificity of 99%.

### 2.3. Statistical Analysis

A Shapiro–Wilk test was used to test the age distribution normality. Differences between groups of categorical variables were assessed using the Chi-square test. Odds ratio ± 95% confidence interval was used to assess the univariate and multivariate association of positive serological tests and explanatory variables (age, area of residence, geographic region, and obstetric history). The level of statistical significance was *p* < 0.05. Statistical analysis was performed using the Web Social Science Statistics program (https://www.socscistatistics.com/, accessed on 20 November 2023) and MedCalc Statistical calculator (2024 MedCalc Software Ltd., https://www.medcalc.org/calc/, accessed on 2 December 2023). STATA/MP 17.0 for Windows (StataCorp LLC, Lakeway, Drive, College Station, TX, USA) was used for a logistic regression analysis.

## 3. Results

The mean age of pregnant women included in the study was 31.5 ± 5.0 (range 16–45) years. The overall IgG seroprevalence rates were 466 (69.9%) (95%CI = 66.2–73.3) for HSV-1 and 25, (3.8%) (95%CI = 2.4–5.5) for HSV-2. Seroprevalence rates according to participants’ characteristics are presented in Table 1 and Table 2.

**Figure 2:**
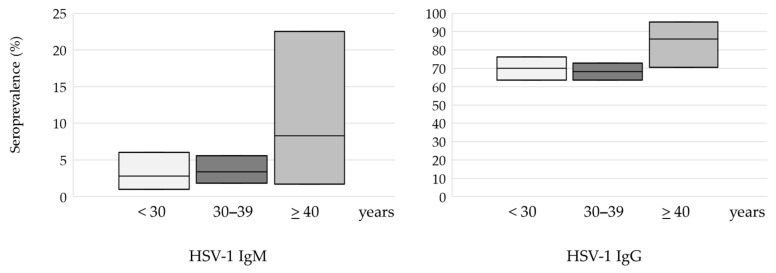
HSV-1 seroprevalence according to age: plots represent seroprevalence (%) with a 95% confidence interval.

**Figure 3:**
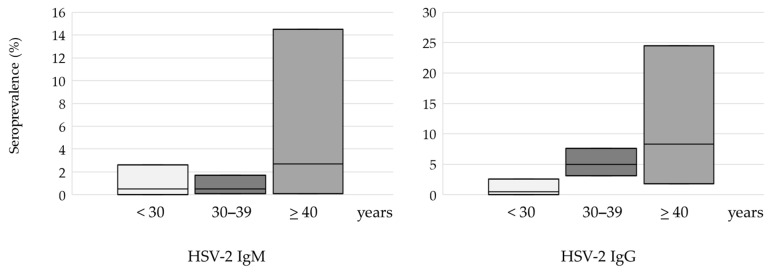
HSV-2 seroprevalence according to age: plots represent seroprevalence (%) with a 95% confidence interval.

In the age groups of <30 and 30–39 years of age, the seroprevalence of HSV-1 was 70.0% and 68.3%, respectively. However, in the population >40 years of age, an increase in seropositivity was recorded to 86.1%; however, this difference did not reach statistical significance (*p* = 0.083) (Figure 2). Seroprevalence of HSV-2 increased gradually with age from 0.5% in participants under 30 years to 8.3% in participants above 40 years, with a significant difference between age groups (*p* = 0.005) (Figure 3).

Participants from urban areas were more often seropositive than participants from suburban and rural areas for both HSV-1 (70.5 vs. 62.3%) and HSV-2 IgG (4.1 vs. 0%) but without a significant difference (*p* = 0.208, and *p* = 0.134, respectively).

Both HSV-1 and HSV-2 seroprevalence rates were slightly higher among participants who lived in continental areas (HSV-1: 70.8%, HSV-2: 4.1%) than those who lived in coastal areas (HSV-1: 67.3%, HSV-2: 2.7%) but without a statistically significant difference (*p* = 0.390 vs. *p* = 0.387).

There was no significant difference in HSV-1 seroprevalence between women with a normal pregnancy (69.5%) and women with an unfavorable obstetric history (70.1%, *p* = 0.883). Women with an unfavorable obstetric history had slightly higher HSV-2 seroprevalence (5.1%) compared to those with a normal pregnancy (3.5%), but this difference was not statistically significant (*p* = 0.193).

The prevalence of acute/recent infections (IgM positive) was 23 (3.5%) (95%CI = 2.2–5.1) for HSV-1 and 4 (0.6%) (95%CI = 0.2–1.5) for HSV-2. No significant difference was observed in the prevalence of HSV-1 or HSV-2 acute infections regarding age (HSV-1: 2.8–8.3%, HSV-2: 0.5–2.7%), urban and suburban/rural area of residence, continental/coastal geographic region, and women with normal pregnancy/unfavorable obstetric history (Table 1 and Table 2).

The results of the risk analysis (Table 3, Table 4 and Table 5) showed that older age was a significant risk factor for HSV-2 IgG seropositivity, while a marginal significance was observed for HSV-1 IgG seropositivity. No significant risk for the HSV-1 and HSV-2 IgG seroprevalence was observed regarding the area of residence, geographic region, and obstetric history.

Analyzing the prevalence of acute infections, age, area of residence, and obstetric history were not found to be associated with either HSV-1 or HSV-2 IgM seropositivity.

## 4. Discussion

Data on HSV prevalence in pregnant women are limited because infection is often asymptomatic and routine HSV screening is not recommended. In addition, genital HSV infection is not a reportable disease [16]. Since data on HSV seroprevalence in Croatia are scarce, we conducted a seroepidemiological study among pregnant women to identify seronegative women who are at risk of contracting primary infection during pregnancy.

The results of this study showed that a significant proportion of pregnant women are HSV-1 seropositive (69.9%), while only 3.8% are seropositive to HSV-2. Comparing the results in this study with the previous Croatian studies, the HSV-1 seroprevalence showed a declining trend from 78.7% to 70.6% (2005 to 2010) and remained stable thereafter (69.9%). However, a continuously declining trend in the HSV-2 seropositivity was observed. In the present study, the HSV-2 seropositivity was 3.8%, compared to 6.8% in 2005–2009 [13] and 8.5% in 2008–2010 [14].

The HSV-1/HSV-2 seroprevalence rates among pregnant women differ within European countries. There are several studies published in the 1990s and 2000s; however, more recent data are lacking. In the 1990s, a seroepidemiological study on the HSV-2 seroprevalence was conducted among pregnant women in Germany. Serum samples were collected in 1988–1989, 1990–1991, and 1996–1997, with seroprevalence of 8.3, 6.3, and 8.9%, respectively [17]. A study from Estonia analyzed the HSV seroprevalence in samples collected from first-trimester pregnant women between February and May 2000 and found seropositivity of 88% for HSV-1 and 24% for HSV-2 [18]. In a study from Lausanne, Switzerland (2004–2007), the first sample was taken in the first trimester, and the second one 6–8 weeks postpartum in seronegative women to assess the incidence of seroconversion (primary infections). HSV-1 seroprevalence was 79.4%, while HSV-2 seroprevalence was 21.2%. A single HSV-1 seroconversion was detected in a patient with symptoms consistent with primary genital herpes [19]. In a study conducted in France (2004), HSV seropositivity was analyzed in pregnant women attending the last routine visit before delivery in an East Paris suburban area. HSV-1 and HSV-2 seroprevalences were 88.2% and 26.3%, respectively. Notably, all women who tested positive for HSV-2 also tested positive for HSV-1. In addition, 6.6% of women were positive for HSV DNA [20]. An Italian study analyzed the HSV-1 and HSV-2 seroprevalence in Bari from 2003 to 2005. Among pregnant women tested, 91.2% and 9.9% showed antibodies to HSV-1 and HSV-2, respectively, while 8.8% were seronegative for both viruses [9]. Similar seroprevalence rates of HSV-1 (94.7%) and HSV-2 (8.2%) were detected in Turkish asymptomatic pregnant women admitted to routine control gynecology and obstetrics clinics of Izmir [21]. One study from Romania (2005) analyzed the HSV seroprevalence among pregnant women, showing anti-HSV-1 IgG antibodies in 87.3% and HSV-2 IgG antibodies in 15.1% of pregnant women in the Bucharest area. Risk factors for HSV-2 seropositivity were a lower level of education and a greater number of sexual partners [22]. Lower seropositivity of HSV-1 was found in Finnish pregnant women tested in 2000 (46.8%), while HSV-2 seropositivity was 9.3% [10]. The other cross-sectional study from Finland tested pregnant women at three time points (1992, 2002, and 2012). Over 20 years, the seroprevalences decreased continuously, HSV-1 from 69.5% to 45%, and HSV-2 from 17.55 to 11% [23]. A very recently published systematic review on the epidemiology of HSV-2 in Europe showed that HSV-2 seroprevalence in Europe has been decreasing by 1% per calendar year over the past 30 years. Furthermore, the results suggested an increasing trend in the incidence of neonatal herpes in Europe in contrast to the decreasing trend of HSV-2 seroprevalence, perhaps reflecting the increasing trend of genital herpes caused by HSV-1 in this region, and increasing maternal age associated with higher HSV-2 seroprevalence [15].

A wide variation in the HSV seropositivity was also observed in other regions of the world. In a study conducted in the USA between 1999 and 2014, the HSV-1 seroprevalence was 59.3% and the HSV-2 seroprevalence was 21.1%, with a stable trend in 1999–2006 and 2007–2014 [16]. In Brazil (Rio de Janeiro, 2015–2016), the HSV-1 and HSV-2 prevalences among pregnant women were 84.5% and 5.6%, respectively [24]. HSV-2 seroprevalence was analyzed in many studies, ranging from 5.8% in Iran [25], 8.7% in India [26], 17.3% in Canada [27], and 23.56% in China [28] to 32.1% in Ethiopia [29]. In one Turkish study (Adana, 1999–2001), a very high HSV-2 IgG seropositivity of 63.1% was detected in asymptomatic pregnant women [30].

In the present study, we found no significant difference in the HSV-1 and HSV-2 seropositivity according to the geographic region, although seropositivity was slightly higher in participants residing in continental regions compared to the participants residing in coastal regions (HSV-1 70.8% vs. 67.3%; HSV-2 4.1% vs. 2.7%). Some countries showed significant regional differences in the HSV seropositivity due to the multiethnic composition of the population. In the Netherlands, a seroepidemiological study was conducted among pregnant women from three cities. Serum samples drawn in the first trimester of pregnancy screening were collected from April to December 1998. Regarding HSV-1, seroprevalence was similar in Amsterdam (73%) and Rotterdam (75%) but significantly higher than in Nijmegen (61%). The seroprevalences of HSV-2 in Amsterdam (35%) and Rotterdam (27%) were also significantly higher than in Nijmegen (11%). Both HSV-1 and HSV-2 seroprevalence rates were higher in non-natives compared to natives [11]. Similar results were observed in France. HSV-2 seropositivity was significantly associated with the country of origin and was 40.5% in African women (sub-Saharan 75%; Maghreb 19%) compared with 9% in women from other countries [20]. Furthermore, in a study conducted in Switzerland, the HSV-1 seroprevalence differed significantly among pregnant women of Swiss and non-Swiss nationality (59.1 vs. 86.4%), while there was no difference in the HSV-2 seroprevalence (20.1 vs. 21.5% [19].

Area of residence (urban, suburban/rural) was not associated with HSV-1 and HSV-2 seroprevalence in the present study. In one previous Croatian study (2005–2009), HSV-1 IgG seropositivity differed according to the area of residence, with rural women more likely to be seropositive (86.0%) compared to urban women (76.4%) [13]. A higher seroprevalence in rural areas could be attributed to the lifestyle of the rural population. People typically live in large families with many children and relatives. Close contact with preschool- and school-aged children is a risk factor for HSV transmission since primary infections usually occur in these cohorts [4]. In contrast, no significant differences in the seroprevalence according to the area of residence were observed for HSV-2 [13]. In the other study (2008–2010), HSV-1 seroprevalence did not differ between participants residing in urban (72.5%) and rural areas (72.6%). Urban place of residence was a significant factor for HSV-2 seroprevalence in univariate analysis but was no longer significant after standardizing for age [14]. In a study from southern Switzerland, HSV-2 seroprevalence was lower in residents of small settlements (<1500 inhabitants), when compared to residents of larger towns [31].

In the present study, the HSV-2 IgG seroprevalence rates were significantly age-related. HSV-2 seropositivity increased progressively from 0.5% in women under 30 years to 8.3% in women 40 years and older. The seropositivity of HSV-1 was stable in pregnant women under 40 years (70.0 and 68.3%), while it was higher in pregnant women above 40 years (86.1%); however, these differences did not reach statistical significance. Similar to our results, maternal age correlated significantly with HSV-2 but not with HSV-1 seropositivity in one Finnish study (South-Western Finland) [10]. In the other study conducted in Finland, increasing HSV-1 and HSV-2 seroprevalence with age was observed [23]. In a Swiss study, the HSV-1 IgG seroprevalence was highest in the age group 15–25 years (84.8%) compared to 26–35-year-olds (76.6%) and 36–45-year-olds (79.5%). The highest seropositivity in the youngest age group may suggest primary HSV infection in this age group. In contrast, a progressive increase in HSV-2 IgG seropositivity (16.8 to 31.8%) was observed with age, similar to the results of our study [19]. The highest HSV-1 IgG seropositivity in the 18–20-year age group (100%) compared to 92.9–96.6% in the age group 21–35 years was also detected in Turkish pregnant women [21]. No association of HSV seroprevalence with age was found in Romanian pregnant women. Neither overall HSV seroprevalence nor type-specific seroprevalence increased statistically significantly with age. High HSV-1 seroprevalence was found even in the youngest age group (88%). HSV-2 seroprevalence was moderate, peaking between 30 and 34 years of age (18.3%) and decreasing slightly thereafter [22].

Regarding the obstetric history, our study found no significant difference in seroprevalence in women with unfavorable obstetric history and women with normal pregnancies (HSV-1 60.1 vs. 69.5%; HSV-2 5.1 vs. 3.5%). Similar results were observed in the previous Croatian studies [13,14]. In contrast, a history of abortion was associated with HSV-2 seropositivity in women in a German study [32]. In addition, in a Hungarian study, a significantly higher HSV-2 seropositivity was found in women attending infertility clinics (12.6%) compared to pregnant women (2.6%) [33]. In a study conducted in Sweden, the overall HSV-2 seroprevalence was 10.4% among pregnant women and 25.2% among female attendees at a clinic for sexually transmitted infections [34].

In our study, the prevalence of primary infections (IgM positive) was 3.5% for HSV-1 and 0.6% for HSV-2. Primary infections occurred in all age groups, with no significant differences. In addition, there was no difference in the prevalence of acute infections in pregnant women with normal pregnancies and with unfavorable obstetric history. Serological screening for HSV infection in symptomatic pregnant women with genital lesions was performed in Turkey from 2008 to 2017. The frequency of HSV IgG seropositivity was 81.3%, and for HSV IgM, it was 4.7% [35].

The heterogeneity of HSV seroprevalence observed in European countries may be partly due to different serological methods (ELISA or WB) for detecting HSV-1 and HSV-2 antibodies. It must be taken into account when interpreting the results. In addition, age, sexual risk behavior, and subregion within Europe could explain a large proportion of the variation in the HSV seroprevalence [14].

There are some limitations of this study that need to be addressed: (a) The retrospective design of the study cannot exclude selection bias; (b) The power of the study is limited by the small number of participants included; (c) A timeline connecting the diagnosis of HSV (seropositivity) to the possible occurrence of obstetric complications cannot be determined. In addition, the small number of pregnant women in the age group above 40 years may influence the marginal difference in the HSV-1 seropositivity between age groups. These limitations should be taken into account when interpreting the results.

Serological screening for HSV in childbearing-aged and pregnant women is important for identifying seronegative women who are susceptible to HSV infection and seropositive women who are at risk for genital herpes recurrence during delivery. In addition, the detection of seronegative women may be clinically important, since subclinical HSV shedding of a seropositive partner may lead to HSV transmission during pregnancy, causing a potential risk for the neonate [23]. Prevention of HSV-2 infection is especially important for women in late pregnancy when the risk of neonatal herpes is highest [5]. In the case of HSV-2 seropositivity, Cesarean section is indicated for women with active genital HSV lesions. Immediate neonatal treatment may be appropriate in the case of vaginal delivery and active infection [17]. If a woman is HSV-2 seronegative and the partner is seropositive, condoms should be used during sexual intercourse in the first and second trimesters of pregnancy. In the third trimester, intercourse should be avoided since condoms do not completely protect against sexual transmission of HSV-2 [36].

## 5. Conclusions

The results of this seroprevalence study show that HSV-1 infection is widely prevalent among pregnant women in Croatia with more than two-thirds (69.9%) infected with this virus. The HSV-2 seroprevalence rate of 3.8% seems to be lower compared to the seroprevalence rates in many European countries. A declining trend in the HSV-2 seroprevalence was observed compared to 2005–2010. Since HSV-1 and HSV-2 are important TORCH pathogens, further seroprevalence studies on a large number of pregnant and childbearing-aged women are needed to identify seronegative women and protect maternal, fetal, and neonatal health.

## Figures and Tables

**Table 1:** Prevalence of HSV-1 antibodies according to sociodemographic characteristics and obstetric history.

Characteristic	N (%) Tested	HSV-1 IgM			HSV-1 IgG		
		N (%)	95%CI	*p*	N (%)	95%CI	*p*
Age group				0.239			0.083
<30 years	214 (32.1)	6 (2.8)	1.0–6.0		150 (70.0)	63.5–76.1	
30–39 years	417 (62.5)	14 (3.4)	1.8–5.6		285 (68.3)	63.6–72.8	
≥40 years	36 (5.4)	3 (8.3)	1.7–22.5		31 (86.1)	70.5–95.3	
Area or residence				0.348			0.208
Urban	614 (92.1)	19 (3.1)	1.9–4.8		433 (70.5)	66.7–74.1	
Suburban/rural	53 (7.9)	4 (7.5)	2.1–18.2		33 (62.3)	47.9–75.2	
Geographic region				0.973			0.390
Continental	483 (72.4)	16 (3.3)	1.9–5.3		342 (70.8)	66.5–74.8	
Coastal	184 (27.6)	6 (3.2)	1.2–6.9		124 (67.3)	60.1–74.1	
Obstetric history				0.461			0.883
Normal pregnancy	453 (67.9)	14 (3.1)	1.7–5.1		315 (69.5)	65.1–73.7	
Unfavorable obstetric history	214 (32.1)	9 (4.2)	1.9–7.8		150 (70.1)	63.5–76.1	

CI = confidence interval.

**Table 2:** Prevalence of HSV-2 antibodies according to sociodemographic characteristics and obstetric history.

Characteristic	N (%) Tested	HSV-2 IgM			HSV-2 IgG		
		N (%)	95%CI	*p*	N (%)	95%CI	*p*
Age group				0.219			0.005
<30 years	214 (32.1)	1 (0.5)	0.0–2.6		1 (0.5)	0.01–2.6	
30–39 years	417 (62.5)	2 (0.5)	0.1–1.7		21 (5.0)	3.1–7.6	
≥40 years	36 (5.4)	1 (2.7)	0.1–14.5		3 (8.3)	1.8–24.5	
Area or residence				0.555			0.134
Urban Suburban/rural	614 (92.1) 53 (7.9)	4 (0.6) 0 (0)	0.2–1.6 0.0–6.7 *		25 (4.1) 0 (0)	2.5–5.8 0.0–6.7 *	
Geographic region				0.230			0.387
Continental	483 (72.4)	4 (0.8)	0.2–2.1		20 (4.1)	2.5–6.3	
Coastal	184 (27.6)	0 (0)	0.0–1.9 *		5 (2.7)	0.9–6.2	
Obstetric history				0.440			0.193
Normal pregnancy	453 (67.9)	2 (0.5)	0.1–1.6		14 (3.5)	1.7–5.1	
Unfavorable obstetric history	214 (32.1)	2 (1.1)	0.2–1.5		11 (5.1)	2.6–9.0	

CI = confidence interval; * one-sided 97.5% confidence interval.

**Table 3:** Univariate risk analysis for HSV-1 seropositivity.

Characteristic	HSV-1 IgM			HSV-1 IgG		
	OR	95%CI	*p*	OR	95%CI	*p*
Age (one-year increase)	1.02	0.94–1.10	0.600	1.03	0.99–1.06	0.052
Urban (Ref.) vs. suburban/rural area of residence	1.69	0.48–5.87	0.406	0.75	0.42–1.35	0.346
Continental (Ref.) vs. coastal geographic region	0.98	0.37–2.55	0.973	0.852	0.59–1.22	0.390
Normal pregnancy (Ref.) vs. unfavorable obstetric history	1.06	0.44–2.51	0.894	1.11	0.78–1.58	0.553

OR = odds ratio; CI = confidence interval.

**Table 4:** Univariate risk analysis for HSV-2 seropositivity.

Characteristic	HSV-2 IgM			HSV-2 IgG		
	OR	95%CI	*p*	OR	95%CI	*p*
Age (one-year increase)	1.09	0.89–1.34	0.380	1.19	1.09–1.30	<0.001
Urban (Ref.) vs. suburban/rural area of residence	1.00	NA	NA	1.00	NA	NA
Continental (Ref.) vs. coastal geographic region	0.22	0.01–4.26	0.323	0.64	0.24–1.75	0.390
Normal pregnancy (Ref.) vs. unfavorable obstetric history	2.12	0.29–15.20	0.451	1.33	0.59–3.00	0.479

OR = odds ratio; CI = confidence interval; NA = not applicable.

**Table 5:** Multivariate logistic regression analysis for the risk of HSV-1 and HSV-2 IgG seropositivity.

Characteristic	HSV-1 IgG			HSV-2 IgG		
	OR	95%CI	*p*	OR	95%CI	*p*
Age (one-year increase)	1.03	0.99–1.06	0.063	1.19	1.09–1.30	<0.001
Urban vs. suburban/rural area of residence	0.77	0.43–1.39	0.396	1	NA	NA
Normal pregnancy vs. unfavorable obstetric history	1.11	0.78–1.58	0.553	1.35	0.59–3.09	0.469

OR = odds ratio; CI = confidence interval; NA = not applicable.

## Data Availability

Data are contained within the article.
